# Epidemiology of Hip Fractures Due to Falls

**DOI:** 10.3390/medicina59091528

**Published:** 2023-08-24

**Authors:** Irena Ilic, Branko Ristic, Ivan Stojadinovic, Milena Ilic

**Affiliations:** 1 Faculty of Medicine, University of Belgrade, 11000 Belgrade, Serbia; 2 Department of Surgery, Faculty of Medical Sciences, University of Kragujevac, 34000 Kragujevac, Serbia; 3 Department of Traumatology, Clinic for Orthopedics and Traumatology, University Clinical Center Kragujevac, 34000 Kragujevac, Serbia; 4 Department of Spinal Surgery, Clinic for Orthopedics and Traumatology, University Clinical Center Kragujevac, 34000 Kragujevac, Serbia; 5 Department of Epidemiology, Faculty of Medical Sciences, University of Kragujevac, 34000 Kragujevac, Serbia

**Keywords:** burden, epidemiology, fall, hip fracture, joinpoint regression analysis, trend

## Abstract

*Background and Objectives:* The epidemiological pattern of the hip fracture burden attributable to falls in Central European countries is still insufficiently known. The aim of this study was to assess the regional and national trends of hip fractures due to falls in Central Europe from 1990 to 2019. *Materials and Methods*: Using the Global Burden of Disease (GBD) 2019 study, this descriptive epidemiological study presents trends in incidence of and Years Lived with Disability (YLDs) from hip fractures due to falls in the region of Central Europe. All estimates (age- and sex-specific rates, and age-standardized rates) were expressed per 100,000. A joinpoint regression analysis was used to assess trends: the average annual percent change (AAPC) with a corresponding 95% confidence interval (95% CI) was calculated. *Results:* Among all new cases of hip fracture in the population as a whole in Central Europe in 2019, 3.9% in males and 7.0% in females were attributable to falls, while the share of hip fractures due to falls in the population aged 70 and over was 16.9% in males and 20.0% in females. About 400,000 new cases of hip fracture due to falls occurred in the Central European region in 2019 (220,000 among males and 160,000 among females), resulting in 55,000 YLDs (32,000 in females and 22,000 in males). About one-third of all new cases (59,326 in males and 72,790 in females) and YLDs (8585 in males, and 10,622 in females) of hip fractures due to falls were recorded in Poland. From 1990 to 2019, the age-standardized incidence rates of hip fracture due to falls showed a decreasing tendency in females (AAPC = −1.1%), and an increasing tendency in males (AAPC = 0.1%). Both in males and females, YLDs rates of hip fracture due to falls in the Central European region decreased (AAPC = −1.6% and AAPC = −2.4%, respectively). *Conclusions:* Hip fracture due to falls represents an important health issue in the Central European region, although incidences and YLDs declined in the most recent decades. However, further efforts to reduce the burden of hip fractures attributed to falls are needed.

## 1. Introduction

Hip fracture represents one of the major health issues worldwide, particularly in the elderly population [1,2,3,4,5]. Globally, the annual incidence of hip fractures was 1.3 million in 1990, with a predicted rise to 3 to 11 million by 2030, and with a prediction to rise to 5 to 21 million by 2050, depending on the assumptions for the estimate [6]. The marked rise in the incidence of hip fracture is primarily considered a result of population growth and increased life expectancy with lifestyle changes that favor osteoporosis [4,5,6]. Many studies have shown that hip fractures represent a remarkable burden to health care systems and societies worldwide, and that patients with hip fracture incur a substantial rise in direct care costs and secondary costs (for specific services and treatments), and have a higher mortality rate than their counterparts [7,8,9]. Recent studies suggest a mortality rate of 17% to 25% within 1 year following hip fracture or surgery in older adults [10,11]. Furthermore, frequent consequences of hip fracture due to falls in older people include reduced functions and abilities (for walking and for basic activities of independent daily living), and compromised quality of life [12]. Some cohort studies indicate that 40% to 60% of surviving patients recovered their pre-fracture level of mobility within 1 year, while 20–60% of subjects who were independent in self-care pre-fracture required assistance for various tasks 1 and 2 years after the fracture [12,13]. Hip prostheses can restore joint function and ability and quality of life [14]. As the number of hip replacements increases every year [15], some recent studies have indicated several potential improvements in the design of prostheses [16]. In the Netherlands, the total cumulative number of Years Lived with Disability due to femoral neck and trochanteric fractures was 24,764 years between 2000 and 2019 [17].

There is a wide geographic variation in hip fracture burden across the world, with higher hip fracture incidence observed in industrialized countries (northern Europe, the US) compared to developing countries in Latin America and Africa [18]. A retrospective analysis of fall-related hip fracture hospitalizations of people aged ≥65 years in Australia showed a trend declining by 2.9% annually [9]. Conversely, a continuous rise in hip fracture rates was reported in developing areas, which could be due to urbanization, obesity, or changes in bone mineral density. However, the background of these divergent secular patterns of the incidence of hip fracture is still not fully elucidated.

According to the World Health Organization, a fall is defined as an event that results in a person returning to rest inadvertently on the ground, floor, or other lower level [19]. Falls were noted to be the second leading cause of death due to unintentional injuries in 2017 [20]. Globally, about one-third of people aged 65 and over fall at least once a year, with the majority of fatal falls occurring in developing countries [20]. The main physical consequences of falls, particularly in older people, are hip fractures [19]. The consequences of a fall and the fear of falling can have a large impact on the quality of life.

The majority of hip fractures are the result of a fall, especially among older persons [21,22]. One study in Amsterdam reported that older age (over 65 years) and female gender are risk factors for fall-related injuries [23]. In England, the hip fracture incidence was relatively higher in more deprived areas, particularly among men, but with no clear pattern among women [24]. In 1990–2019, falls tended to be the largest cause of injury across all European regions [25]. The epidemiological patterns of hip fractures attributable to falls in the Central European countries are still insufficiently known. This study aimed to assess trends in the disease burden of hip fractures due to falls from 1990 to 2019.

## 2. Methods

In this study, we used the latest data from the Global Burden of Disease (GBD) 2019 study to estimate the trends of hip fracture burden due to falls in Central Europe [26].

### 2.1. Study Design

This study was carried out using a descriptive epidemiological method. Additionally, in this work, design of an ecological study was incorporated.

### 2.2. Study Population

Based on the GBD 2019 study, the world is separated into 21 regions (one of them is the region of Central Europe) according to epidemiological similarities and geographical proximity. According to the GBD 2019 study, the Central European region includes 13 countries (Albania, Bosnia and Herzegovina, Bulgaria, Croatia, Czechia, Hungary, North Macedonia, Montenegro, Poland, Romania, Serbia, Slovakia, and Slovenia) comprising about 115 million inhabitants [26].

### 2.3. Data Source

The annual number of hip fracture cases attributable to falls from 1990 to 2019, categorized by year, age, sex, and country, was extracted from the GBD 2019 study [26]. GBD 2019 provides a comprehensive assessment of the all-cause and cause-specific burden for 369 diseases and injuries and 87 risk factors across the world from 1990 to 2019. GBD 2019 uses all available disease and injury data from a range of data source types, including vital statistics, household surveys, registries, and hospital records. Based on the International Classification of Diseases (ICD), for the classification of hip fracture, code M84.459A (Pathological fracture, hip, unspecified …… initial encounter for fracture) was used. Based on the ICD, for the classification of falls, codes W00–W19.9 (fall due to ice and snow; on same level from slipping, tripping and stumbling; other fall on same level due to collision with another person; while being carried or supported by other persons; from non-moving wheelchair, non-motorized scooter and motorized mobility scooter; from bed; from chair; from other furniture; on and from playground equipment; on and from stairs and steps; on and from ladder; on and from scaffolding; from, out of or through building or structure; from tree; from cliff; fall, jump or diving into water; other fall from one level to another; other slipping, tripping and stumbling and falls; unspecified fall) were used. The GBD estimates have been developed and methods appropriately documented following the Guidelines for Accurate and Transparent Health Estimates Reporting [27].

### 2.4. Measures

Age- and sex-specific rates, as well as age-standardized rates (ASRs) for incidence and Years Lived with Disability (YLDs) caused by hip fractures due to falls, were presented. All rates were expressed per 100,000 persons. The age-standardized rates were calculated by using the world standard population developed for the GBD study, using the direct standardization method. To investigate the age patterns of incidence and YLDs, data were categorized into 5 age groups: 0–9 years, 10–24, 25–49, 50–69, and  ≥ 70 years. The analyses were performed for males and females separately. In this study, incidence and YLDs were presented overall and separately by countries. The YLDs is a measure of the burden of living with a disease or disability, i.e., years of healthy life lost due to disability. The YLDs are expressed in the number of years (one YLD represents the equivalent of one year of healthy life lost due to disability or disease) [26].

The Human Development Index (HDI) represents a composite index, measuring average achievement in three key dimensions of human development: a long and healthy life (assessed by life expectancy at birth), knowledge (measured by mean of years of schooling for adults aged 25 years and more), and a decent standard of living (measured by gross national income per capita) [28]. The value of the HDI ranges from 0 to 1, where 1 indicates the highest, ideal development. Data about HDI at the national level were extracted from the United Nations Development Programme.

### 2.5. Statistical Analysis

In this study, incidence and YLDs, by age, sex, country, HDI, and over time were compared. The size and course of temporal trends were evaluated using the joinpoint regression analysis (Joinpoint regression software, Version 4.9.0.0; National Cancer Institute, Bethesda, MD, USA—March 2021, available through the Surveillance Research Program of the US National Cancer Institute) [29]. The joinpoint regression analysis identified points called joinpoints, which denote a statistically significant change in a trend and determine the trends between joinpoints. The permutation test was applied using the Monte Carlo method. The grid search was used to fit the segmented regression function [30]. Using the calendar year as the regression variable, the joinpoint regression analysis estimated the average annual percent change (AAPC) as a summary measure of the trend, with the corresponding 95% confidence interval (95% CI) [31]. In this study, the terms “significant increase” or “significant decrease” were used, in order to signify that the slope of the trend was statistically significant (*p* < 0.05, on the basis of the statistical significance of the AAPC compared to zero). Disparities in trends according to sex and age were tested using a comparability test, i.e., a test of coincidence and a test of parallelism [32]. A *p* value < 0.05 was considered statistically significant for all tests.

The correlation between the incidence and YLDs from hip fracture attributed to falls and the HDI was estimated with the Pearson correlation coefficient. Statistical analyses were conducted using SPSS Software (version 20.0, Chicago, IL, USA).

### 2.6. Ethics Statement

This study was approved by the Ethics Committee of the Faculty of Medical Sciences, University of Kragujevac (No. 01–14321). The study was conducted using publicly available data and data are fully aggregated and anonymized.

## 3. Results

**Figure 1:**
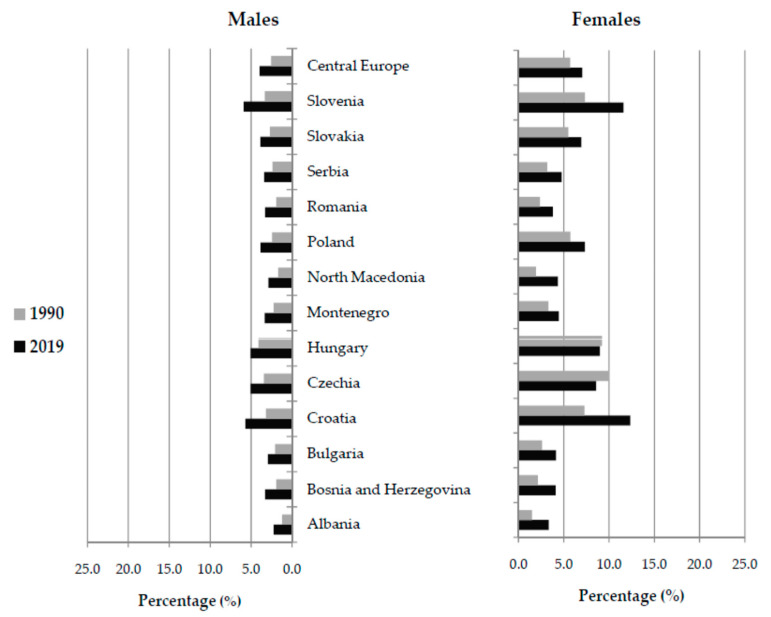
The proportion (%) of new cases of hip fracture due to falls (of all new cases of hip fracture) in the Central European region, by country and sex, in 1990 and 2019 [26].

Of all new cases of hip fracture in Central Europe in 2019, 3.9% in males and 7.0% in females were attributable to falls (Figure 1). In males, the proportion of new cases of hip fracture due to falls was higher in 2019 than in 1990 in all countries in Central Europe. In females in Czechia and Hungary, the observed proportion of new cases of hip fracture due to falls in all new cases of hip fracture was lower in 2019 than in 1990. The highest percentage of hip fractures due to falls was evident in females in Croatia (12.4% of the total), followed by Slovenia (11.6%) in 2019.

**Figure 2:**
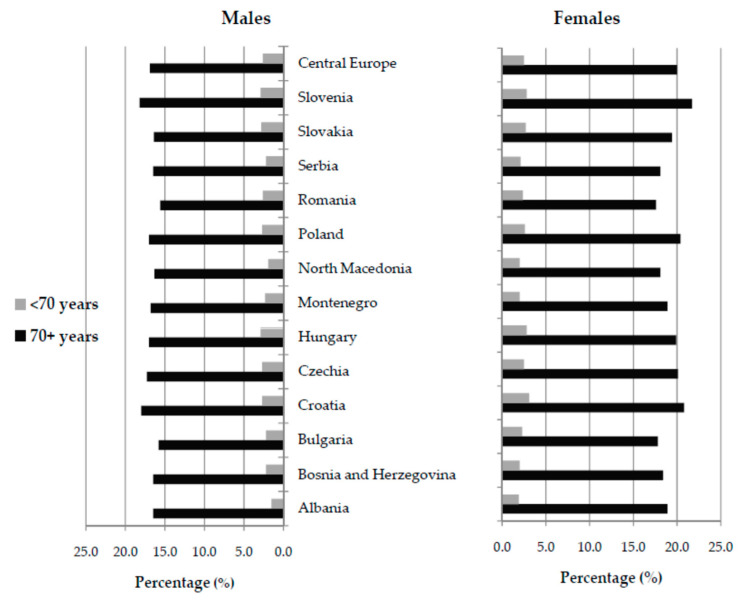
The proportion (%) of new cases of hip fracture due to falls (of all new cases of hip fracture) in the Central European region, by country, sex, and age, in 2019 [26].

Of all new cases of hip fracture in the population aged 70 and over in Central Europe in 2019, 16.9% in males and 20.0% in females were attributable to falls (Figure 2). Of all new cases of hip fracture in the population under the age of 70 in Central Europe in 2019, 2.6% in males and 2.5% in females were attributable to falls. In 2019, in males aged 70 and over, the observed proportion of new cases of hip fracture due to falls in all new cases of hip fracture was the highest in Slovenia and Croatia (about 18.0%). Also, in those aged 70 and over, the highest percentage of hip fractures due to falls was evident in females in Slovenia (21.7% of the total), followed by Croatia (20.8%) in 2019.

**Figure 3:**
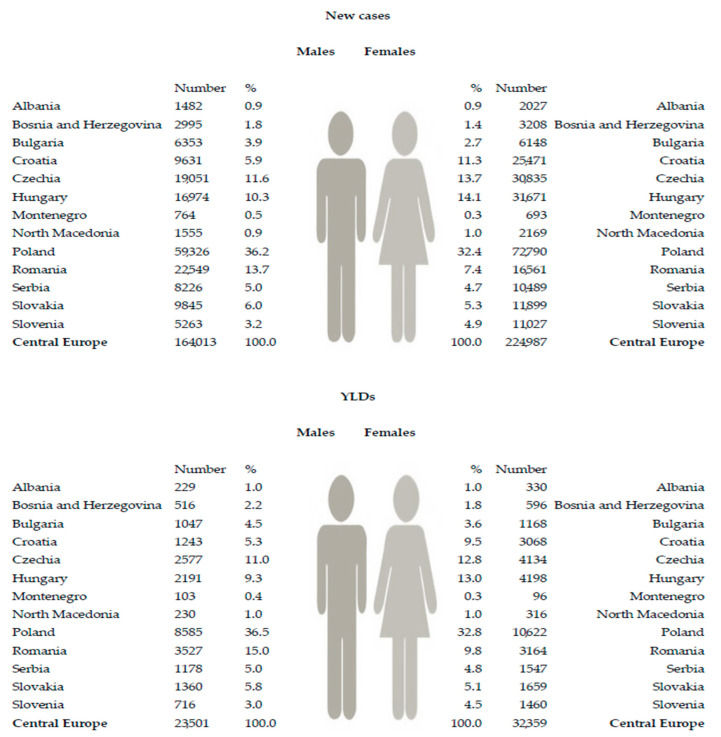
Numbers of new cases and Years Lived with Disability (YLDs) of hip fracture due to falls in the Central European region, by country and sex, in 2019 [26].

A total of about 400,000 new cases of hip fracture due to falls occurred in the Central European region in 2019 (220,000 males and 160,000 females), resulting in 55,000 (32,000 in females and 22,000 in males) YLDs due to hip fracture attributable to falls in 2019 (Figure 3). About one-third of all new cases and YLDs from hip fractures due to falls were recorded in both men and women in Poland. The lowest percentage (≤1.0% of all) of new cases and YLDs from hip fractures due to falls in both females and males was reported in Albania.

**Figure 4:**
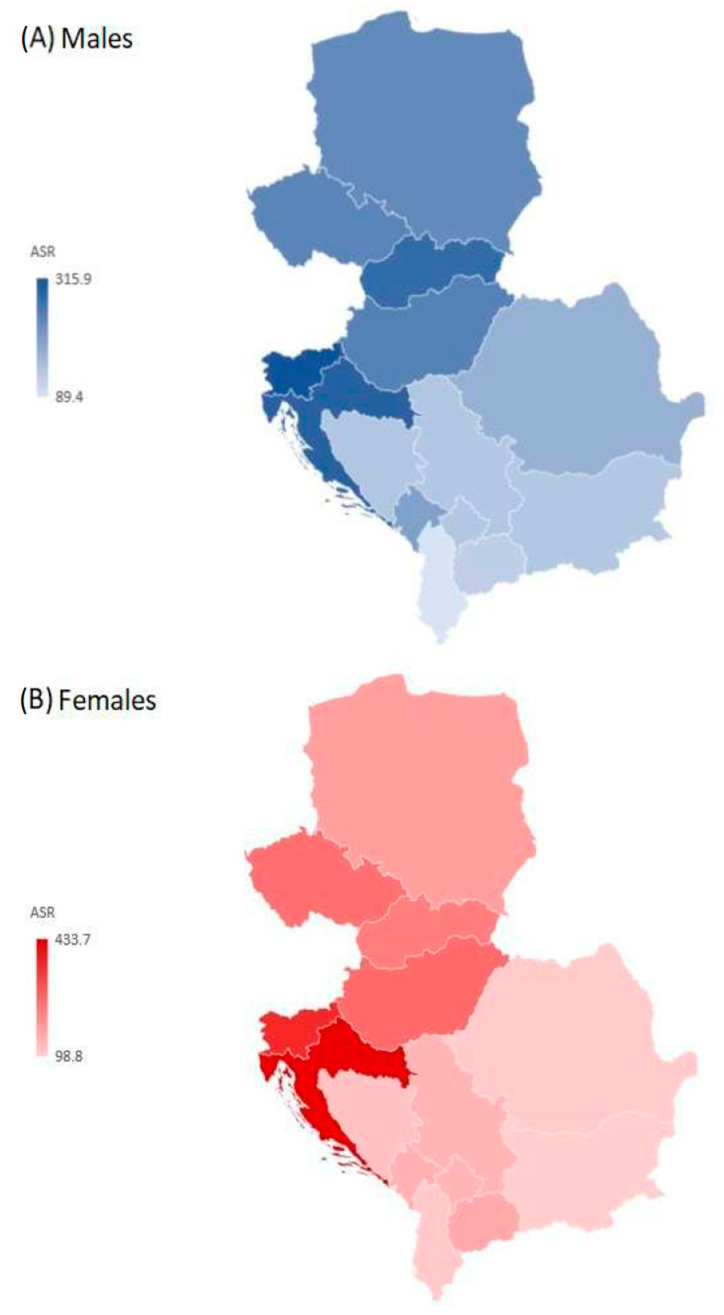
Incidence of hip fracture due to falls in the Central European region, by country and sex, in 2019. ASR = Age-standardized rates (per 100,000) [26].

There were significant variations in the incidence and YLDs from hip fractures attributed to falls between countries by sexes in 2019. The regional age-standardized rate of incidence of hip fracture due to falls was 208.0 per 100,000 persons in males and 186.2 per 100,000 in females in 2019 (Figure 4). There was an approximately fourfold difference between the countries with the highest and lowest age-standardized rates of incidence of hip fracture attributed to falls, with the highest rates in males in Slovenia (315.9 per 100,000) and in females in Croatia (433.7 per 100,000), and the lowest rates in males in Albania (89.4 per 100,000) and in females in Bulgaria (98.8 per 100,000) in 2019.

**Figure 5:**
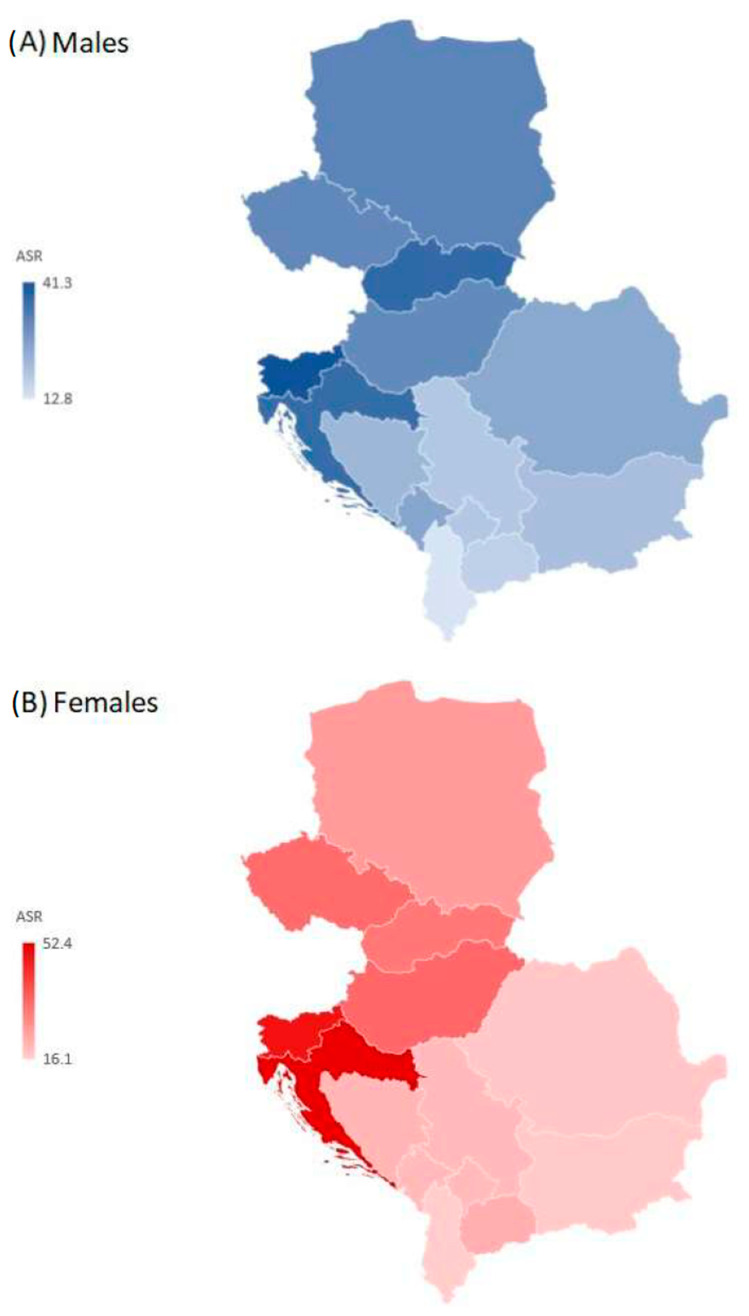
Years Lived with Disability (YLDs) of hip fracture due to falls in the Central European region, by country and sex, in 2019. ASR = Age-standardized rates (per 100,000) [26].

The regional age-standardized rate of YLDs from hip fractures due to falls was 28.3 per 100,000 persons in males and 26.1 per 100,000 in females in 2019 (Figure 5). There was a nearly fourfold difference between the countries with the highest and lowest age-standardized rates of YLDs from hip fracture attributed to falls, with the highest rates in males in Slovenia (41.3 per 100,000) and in females in Croatia (52.4 per 100,000), and the lowest rates in both males and females in Albania (12.8 per 100,000 and 16.1 per 100,000, respectively) in 2019.

**Figure 6:**
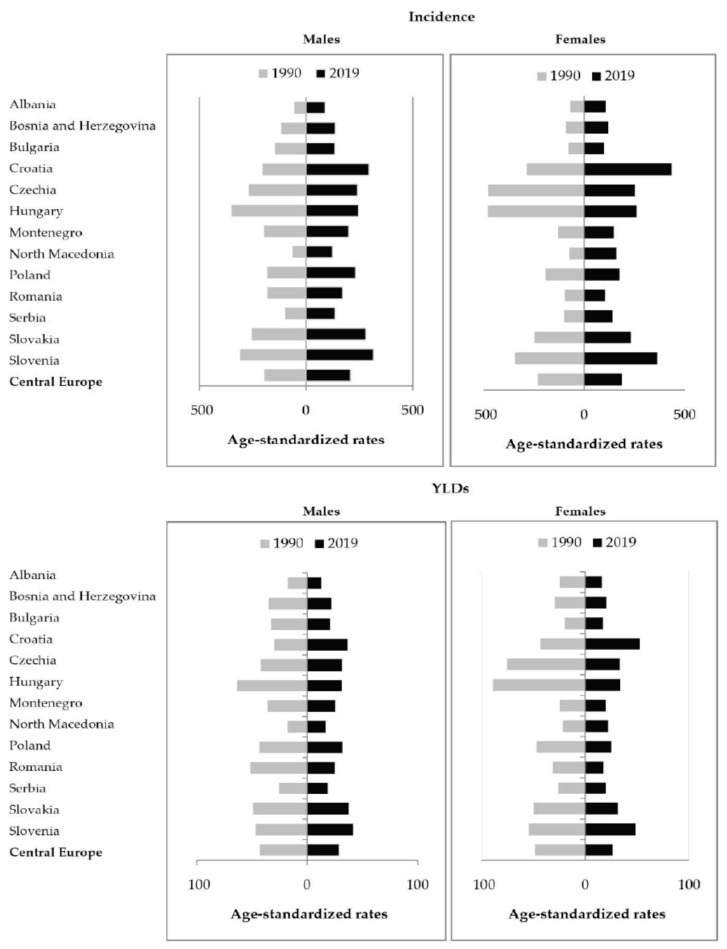
Incidence and Years Lived with Disability (YLDs) of hip fracture due to falls in the Central European region, by country and sex, in 1990 and 2019. Age-standardized rates per 100,000 [26].

Except for the ASRs of incidence in males only, the differences in the ASRs of incidence in females and YLDs in both sexes for hip fracture due to falls were not striking over the studied period. In comparison to males, the ASRs of incidence and YLDs from hip fractures due to falls in the Central European region were lower in females in 2019 (Figure 6). In males in the Central European region, it was observed that the ASR of incidence of hip fracture due to falls was only higher in 2019 than in 1990 (208.0 per 100,000 versus 195.6 per 100,000 respectively). In contrast, the ASRs of incidence of hip fracture due to falls in females, as well as the ASRs for YLDs in both sexes, were lower in 2019 than in 1990.

**Figure 7:**
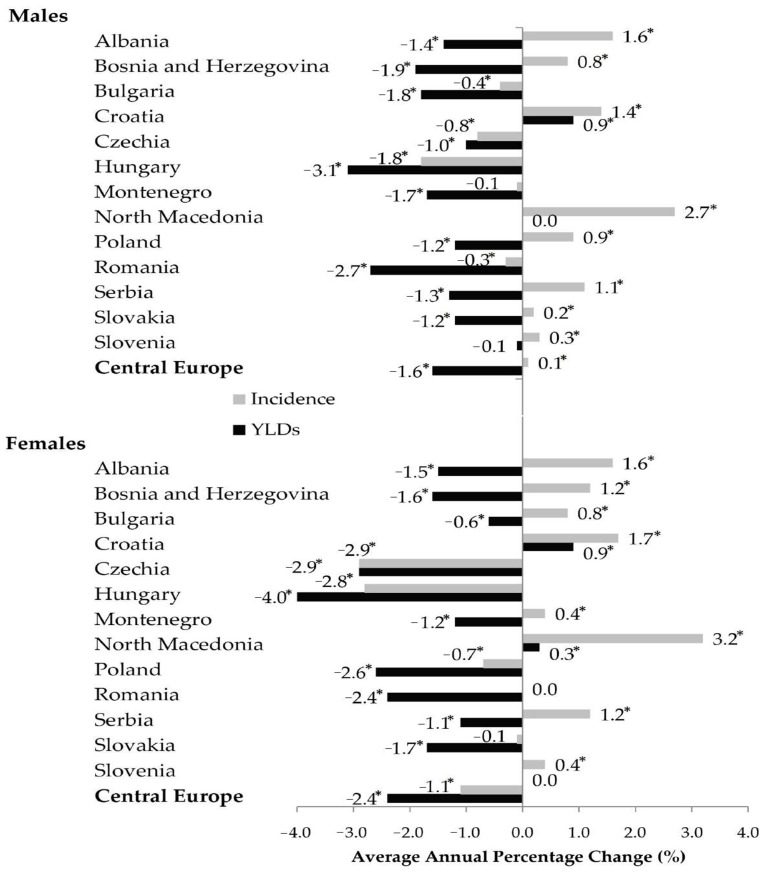
Trends in age-standardized rates (per 100,000) in incidence and Years Lived with Disability (YLDs) of hip fracture due to falls in the Central European region, by country and sex, 1990–2019: a joinpoint regression analysis. * Statistically significant trend; for full period presented AAPC (average annual percent change) [26].

Regionally, from 1990 to 2019, the age-standardized incidence rates of hip fracture due to falls had a decreasing tendency in females (AAPC = −1.1%), and an increasing tendency in males (AAPC = 0.1%) (Figure 7). Both in males and females, YLDs rates of hip fracture due to falls in the Central European region decreased by AAPC = −1.6%, and AAPC = −2.4%, respectively (Figure 7, Table 1). According to the comparability test, trends of incidence and YLDs from hip fractures due to falls in males and females were not parallel and not coincident (*p* < 0.05).

With the exception of Croatia, where a trend of increasing YLDs (AAPC = 0.9%) for hip fracture due to falls in males was recorded, decreasing trends in YLDs rates were recorded in males in all other countries in the Central European region. With the exception of Croatia and North Macedonia, where trends of increasing YLDs (AAPC = 0.9% and AAPC = 0.3%, respectively) for hip fracture due to falls in females were recorded, decreasing trends of YLDs rates were recorded in females in all other countries in the Central European region.

Trends in both incidence and YLDs rates of hip fracture due to falls were decreasing in males in Bulgaria, Czechia, Hungary, Montenegro, and Romania. Trends in both incidence and YLDs rates of hip fracture due to falls were decreasing in females in Czechia, Hungary, Poland, and Slovakia.

Both in males and females, unfavorable trends in both incidence (AAPC = 1.4%, and AAPC = 0.9%, respectively) and YLDs rates (AAPC = 1.7%, and AAPC = 0.9%, respectively) of hip fracture due to falls were reported only in Croatia.

Both in males and females, favorable trends in both incidence and YLDs rates of hip fracture due to falls were reported in Czechia (in males: AAPC = −0.8% in incidence rates, and AAPC = −1.0% in YLDs rates; in females: AAPC = −2.9% in incidence rates, and AAPC = −2.9% in YLDs rates) and Hungary (in males: AAPC = −1.8% in incidence rates, and AAPC = −3.1% in YLDs rates; in females: AAPC = −2.8% in incidence rates, and AAPC = −4.0% in YLDs rates).

Incidence and YLDs rates of hip fracture due to falls increased with age both in males and females in the Central European region (Table 1). In males and females in the younger age groups (0–9 years and 10–24 years), the differences in rates of incidence of and YLDs from hip fractures due to falls were not striking. In the middle-aged persons group (25–49 years and 50–69 years), rates of incidence and YLDs were almost two times higher in males than in females. In the oldest age group (70+ years), rates of both incidence and YLDs were higher in females than in males. In females, the incidence rate of hip fracture due to falls was almost 9 times higher in people aged 70 or older than in people aged 50–69 years, while in males this difference was smaller (3-fold). While in the youngest group (0–9 years), similar trends of increase in the incidence rates of hip fracture due to falls in both sexes were recorded, in the oldest age groups (50–69 years and 70+ years), opposite trends were recorded, i.e., trends of significant increase in incidence in males versus significant trends of decrease in incidence in females. According to the comparability test, trends in the incidence of hip fracture due to falls by age were not parallel (*p* < 0.05), neither in males nor in females.

Significantly decreasing trends of YLDs rates were observed in both sexes in all age groups, except in females in the youngest group (0–9 years), where a significantly increased trend was recorded (AAPC = 0.4%).

**Figure 8:**
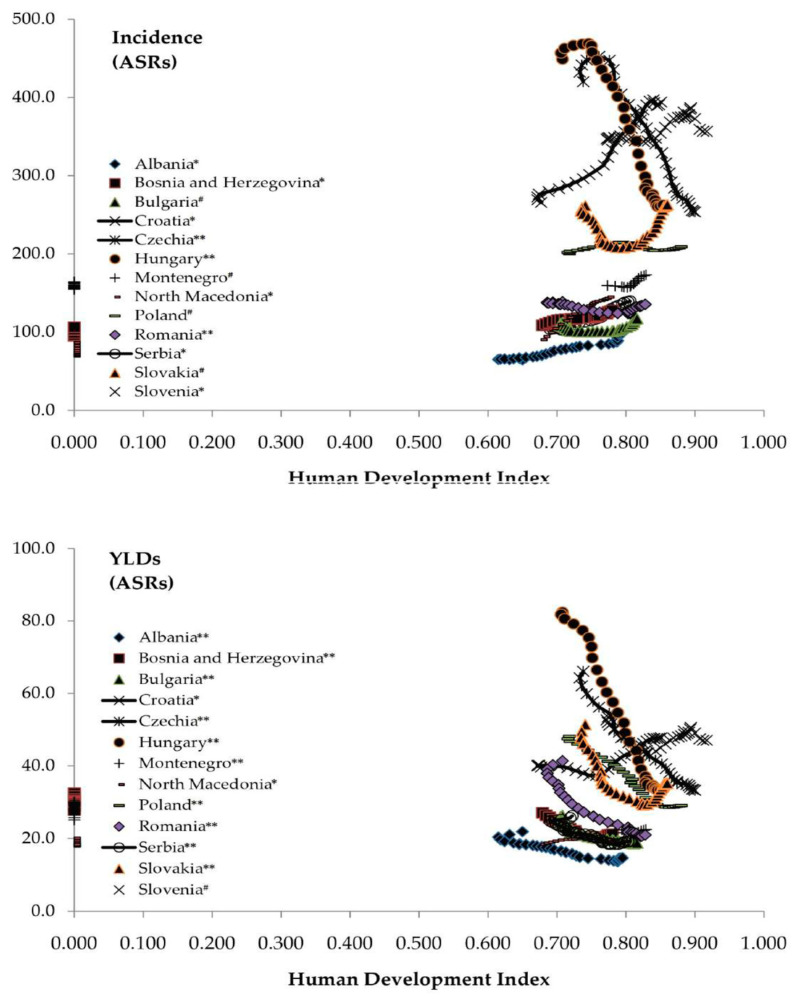
Correlation of the age-standardized rates (per 100,000) in incidence and Years Lived with Disability (YLDs) of hip fracture due to falls in the Central European region with Human Development Index, by country, during 1990–2019.* Statistically significant positive correlation; ** statistically significant negative correlation; ^#^ absence of correlation [23,28].

The Pearson coefficient showed a significant positive correlation between the incidence of hip fracture due to falls and HDI in Albania, Bosnia and Herzegovina, Croatia, North Macedonia, Serbia, and Slovenia in 1990–2019 (equally at *p* < 0.001) (Figure 8). A negative correlation between the incidence and HDI was observed in Czechia, Hungary, and Romania (equally at *p* < 0.001), and an absence of correlation with HDI in Bulgaria, Montenegro, Poland, and Slovakia (equally at *p* > 0.05). A significant positive correlation was reported between YLDs from hip fractures due to falls and the HDI in Croatia and North Macedonia in 1990–2019 (equally at *p* < 0.001), with an absence of correlation in Slovenia (r = −0.223, *p* > 0.05), while a significant negative correlation was noted in all other countries in the Central European region (equally at *p* < 0.001).

## 4. Discussion

The burden of hip fracture due to falls varied significantly between the Central European countries. The greatest burden of hip fractures was observed in Croatia and Slovenia for both males and females, while the lowest burden was reported in Albania. The trends in YLDs from hip fractures due to falls were decreasing in both sexes in almost all age groups, correlating with the level of social development measured by the Human Development Index. Unfavorable trends in the frequency of hip fractures due to falls in the youngest age group in both sexes deserve special attention.

Previous studies have noted marked geographic variations in the incidence of hip fracture, with the highest rates observed in the Nordic countries, followed by North America and Europe [18,25,33]. Globally, the lowest incidence rates of hip fracture are reported in Africa and Asia, which some authors attribute to underregistration, low urbanization, and a younger population as a whole. This study showed that the highest rates of incidence of and YLDs from hip fractures due to falls were observed in both sexes in Slovenia and Croatia in 2019. On the other hand, in the Central European region in 1990, the highest rates of incidence of and YLDs from hip fractures due to falls were observed in both sexes in Hungary and Czechia. In addition to the increase in the share of the elderly population, a birth cohort effect and a country’s development level could also play a role in the rates of incidence of and YLDs from hip fractures: all countries in the Central European region were part of the former Eastern European socialist bloc in the past, and in the 1990s they were at different levels of social development and circumstances, whereby differences in the lag period of changes in society resulted in differences in the burden of diseases such as hip fracture due to falls in 2019. As a current consequence of different phases in the epidemic process, the rates of incidence of and YLDs from hip fractures due to falls in Croatia and Slovenia in 2019 even surpassed the rates of incidence of and YLDs from hip fractures due to falls recorded in Hungary and Czechia in 1990. Low rates of YLDs from hip fractures due to falls in Hungary and Czechia in 2019 could be attributed to the availability of improved therapy and rehabilitation during the last decades [5,12]. On the other hand, low rates in some other countries in the Central European region in part could be due to underreporting of hip fractures due to falls [34].

After the increasing trends in the 1970s and 1980s, the developed countries in North America (Canada and the USA) and Europe observed decreasing and stable trends in the incidence rates of hip fracture [3,5,25,33]. Contrary to that, the age-standardized rates of incidence for hip fracture due to falls increased from 1990 to 2019 in almost all of the Central European countries in both sexes (except Czechia and Hungary). On the other hand, the age-standardized rates of YLDs from hip fractures due to falls decreased from 1990 to 2019 in almost all of the Central European countries (except Croatia and North Macedonia). Some authors suggested that international variations in hip fracture incidence trends could be related to demographic changes, environmental factors associated with economic development, urbanization, the level of physical activity, obesity, nutritional status, comorbidity, osteoporosis, the use of drugs such as bisphosphonates, latitude, and preventive measures [33,34,35,36,37].

The age- and sex-specific rates of incidence of and YLDs from hip fracture decreased from 1990 to 2019 in almost all of the Central European countries (except Croatia and North Macedonia), which could be attributed to the improvements in the management of disease [37]. Furthermore, rates of incidence of and YLDs from hip fractures due to falls exponentially increased with age in both sexes. In recent decades, there has been a rapid growth of the elderly population who reach the age of 85 years and over, thus creating a population of a usually precarious health, who recover more slowly after injuries and are more likely to have hip fractures due to falls compared to the younger population. Further, some studies showed that older people in the United States had a lower femoral neck bone mass density in the 2005–2006 period than in 1988–1994, indicating that they were at high risk for hip fractures [38]. A recent study in Bulgaria reported similar results [39]. In addition, some authors suggested that changes in trends correlated with changes in environmental and/or intrinsic factors, such as poverty, ethnicity, rurality, changes in body mass index, osteoporosis medication, the plasma level of vitamin D, sun exposure, calcium intake, nutritional status, and physical activity [5,22,23,24]. Also, some reports point to the possible importance of early exposure to environmental factors (primarily dietary factors, physical activity, comorbidities), whereby etiological factors could act earlier in the life course, causing an increase in the rate of hip fractures in later life [40,41].

Similarly to the results of this research, some studies indicated that the development of society and growing wealth in certain areas across the world is followed by changes in trends in rates of incidence of and YLDs from hip fractures due to falls, suggesting that economic prosperity is of great importance for the magnitude and direction of trends of hip fracture [9,24,42]. With the prosperity of society comes better acute care and increased availability of improved therapeutic modalities and preventive strategies, which results in an increase in the number of elderly people who survive after hip fractures due to falls, a significant percentage of whom require long-term care due to various degrees of disability [5,42,43]. In this study, the Human Development Index inversely correlated with rates of YLDs from hip fractures due to falls, suggesting that people in areas with a prosperous social economy and well-being are more likely to be aware of issues concerning hip fractures due to falls, to have a healthy lifestyle, and to have access to higher levels of health care services, which would be beneficial to the prevention and management of hip fractures.

Finally, international comparisons of the rates of incidence of and YLDs from hip fractures due to falls should be made with caution, because in the developing countries, the question of the underestimation of rates due to the lack of national databases can always be raised, as well as the issue of overestimation of rates in developed countries due to more complete databases, extended life expectancy, and improvements in health care due to increased industrialization and urbanization. Future studies are necessary to further evaluate the trends and better understand the reasons for the changes in trends.

### Strengths and Limitations of the Study

There are several limitations to this study. First, the question of data quality can always be raised: some reports showed that there are differences regarding inclusion criteria between different hip fracture registries in European countries, as well as differences in the way indicators are reported [34]. Further, the study design (i.e., a correlation/ecological study design) does not allow us to establish a causal relationship between the occurrence of hip fracture due to falls and the Human Development Index, because we do not have data on well-being at the level of individual cases. Also, the lack of data on other characteristics of the cases (such as socio-economic status, occupation, comorbidities) is a drawback of this study. Finally, due to the delayed availability of much data during the COVID-19 pandemic, the data regarding hip fracture due to falls for the last few years could not be included in this analysis.

## 5. Conclusions

The magnitude of the burden of hip fractures due to falls in the Central European region was considerable in the last decades. Further efforts to explore the patterns of occurrence of hip fractures and to identify the risk factors implicated in this public health issue could be important for disease control.

## Figures and Tables

**Table 1:** Trends * in incidence and Years Lived with Disability (YLDs) rates (per 100,000) of hip fracture due to falls in Central Europe, by age and sex, 1990–2019: a joinpoint regression analysis.

	Males			Females		
Age	Age-Specific Rates		AAPC (95% CI)	Age-Specific Rates		AAPC (95% CI)
	1990	2019		1990	2019	
	Incidence					
0–9	9.1	12.1	+0.9 * (0.7 to 1.1)	10.6	15.6	+1.4 * (1.2 to 1.5)
10–24	15.3	53.3	−0.1 * (−0.2 to −0.0)	35.8	47.0	+1.0 * (0.9 to 1.1)
25–49	179.5	161.3	−0.7 * (−0.8 to −0.5)	70.5	79.5	0.2 (−0.0 to 0.3)
50–69	352.3	414.1	+0.7 * (0.7 to 0.8)	267.1	246.7	−0.7 * (−0.9 to −0.5)
70+	1028.9	1222.7	+0.4 * (0.3 to 0.5)	2536.1	1923.8	−1.2 * (−1.4 to −0.9)
	Age-standardized rates					
All ages	195.6	208.0	+0.1 * (0.0 to 0.2)	230.3	186.2	−1.1 * (−1.2 to −1.0)
	Years Lived with Disability					
0–9	0.7	0.7	−0.1 (−0.4 to 0.1)	0.8	0.9	+0.4 * (0.1 to 0.6)
10–24	6.1	4.1	−1.6 * (−1.8 to −1.4)	4.8	3.8	−0.8 * (−1.1 to −0.6)
25–49	36.4	18.8	−2.8 * (−3.0 to −2.5)	17.5	10.9	−2.1 * (−2.4 to −1.7)
50–69	106.1	68.2	−1.5 * (−1.7 to −1.3)	75.5	44.0	−2.3 * (−2.7 to −2.0)
70+	206.4	175.7	−0.6 * (−0.9 to −0.4)	494.9	266.4	−2.2 * (−2.6 to −1.9)
	Age-standardized rates					
All ages	43.2	28.3	−1.6 * (−1.8 to −1.4)	48.4	26.2	−2.4 * (−2.7 to −2.2)

* Statistically significant trend (*p* < 0.05); AAPC, for full period presented AAPC (average annual percent change); CI = confidence interval [26].

## Data Availability

Data are contained within the article.
